# Multigenerational experimental simulation of climate change on an economically important insect pest

**DOI:** 10.1002/ece3.6847

**Published:** 2020-10-27

**Authors:** David Schneider, Alejandra G. Ramos, Alex Córdoba‐Aguilar

**Affiliations:** ^1^ Departamento de Ecología Evolutiva Instituto de Ecología Universidad Nacional Autónoma de México México Mexico; ^2^ Facultad de Ciencias Universidad Autónoma de Baja California Ensenada Mexico

**Keywords:** climate change, experimental simulation, insect pest, life history, multigenerational, *Phaseolus vulgaris*, reciprocal transplant, *Zabrotes subfasciatus*

## Abstract

Long‐term multigenerational experimental simulations of climate change on insect pests of economically and socially important crops are crucial to anticipate challenges for feeding humanity in the not‐so‐far future. Mexican bean weevil *Zabrotes subfasciatus*, is a worldwide pest that attacks the common bean *Phaseolus vulgaris* seeds, in crops and storage. We designed a long term (i.e., over 10 generations), experimental simulation of climate change by increasing temperature and CO_2_ air concentration in controlled conditions according to model predictions for 2100. Higher temperature and CO_2_ concentrations favored pest's egg‐to‐adult development survival, even at high female fecundity. It also induced a reduction of fat storage and increase of protein content but did not alter body size. After 10 generations of simulation, genetic adaptation was detected for total lipid content only, however, other traits showed signs of such process. Future experimental designs and methods similar to ours, are key for studying long‐term effects of climate change through multigenerational experimental designs.

## Graphical abstract

This multigenerational simulation of climate change demonstrates how an economically important pest of the bean seeds; the Mexican bean weevil, will be leaner, fitter and show genetic resilience under the conditions predicted for 2100.

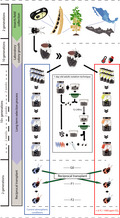

## 1 INTRODUCTION

Current models of human activity and climate change predictions, according to the most pessimistic scenarios, foresee an atmospheric concentration of 1000 ppm of CO_2_ and an associated global temperature increase of 6°C by 2100 (IPCC, 2007, 2014). Assuming that human population growth and food consumption follow its current trend (Bajželj et al., 2014), global crop production will require a 60% increase by mid‐century to respond to food demands (Godfray et al., 2010). Notwithstanding, climate change will hinder this achievement in two ways: (a) crop production will encounter constraints due to plant productivity itself (Olesen et al., 2011; Waha et al., 2013), and (b) pest population dynamics and physiology will be altered (Bale et al., 2002; Harrington et al., 2001). While there is no easy way to deal with future plant productivity constrains, pest management can be adjusted (Estay et al., 2009, 2014). Notwithstanding, we are still far away from understanding how pests will deal with realistic climate change scenarios (Bannerman & Roitberg, 2014; Gillespie et al., 2012; Haridas et al., 2016).

Typical variables associated to global change scenarios, namely increased temperature, and CO_2_, affect pest survival and/or fecundity. For example, development survival (measured from egg‐to‐adult) of *Heliothis viriplaca* (Cui et al., 2018) and *Thrips palmi* (Yadav & Chang, 2014) increased with elevated temperature until reaching a certain threshold. Similarly, development survival, fecundity and parasitizing activity of *Trichogramma buesi,* increased with temperature until a maximal value was reached (Reznik et al., 2009). Additionally, when the bean weevil *Acanthoscelides obtectus* grows at lower altitude, hence at higher temperature, fecundity increased, as well as egg hatching rate and ovarian production (Huignard & Biemont, 1978). These studies have also suggested that although an increase of temperature favors survival and fecundity, there is a temperature threshold that led trait expression start plateauing or collapsing, most likely because a maximum of metabolic rate was reached.

Resource allocation theory assumes that organisms have a limited amount of resources, which will be traded off among life history traits (Boggs, 2009; Deas & Hunter, 2014; Parker & Courtney, 1984; Pianka, 1981; Roff, 2002). For the case of females, a large proportion of their resources must be allocated to producing successful offspring. However, insect oviposition opportunities are often coerced to assign offspring to a limited patch of resources, such as seeds or insect hosts (Díaz‐Fleischer & Aluja, 2003). Egg load, or the number of mature eggs a female is carrying (Ellers & Jervis, 2003; Harvey et al., 2001), is expected to shape the temporal (and spatial) variability in choices related to these oviposition resources. However, given that temperature is a major driver of insect lifespan, one expects oviposition strategy to covary with temperature. Such alteration will take place, for example, if death or loss of ability to reproduce is imminent (Sevenster et al., 1998). In this case, females will produce and lay eggs as soon as possible.

Selection and evolution of thermal reactions imply that environmental temperature and adult body size are linked in different geographical populations (i.e., Bergmann's rule). This relationship predicts that species living in colder conditions reach a larger adult size than species living in hotter climates (Bergmann, 1847). Alternatively, the temperature–size rule stipulates that the plastic phenotypic response to increased temperatures can produce smaller insects by increasing developmental rate (Atkinson, 1994) as increased temperatures shorten insect life span (Papanikolaou et al., 2013). On the other hand, Bergmann's rule implies that environmental temperature and adult body size are linked in different geographical populations: species living in colder conditions reach a larger adult size than species living in hotter climates (Bergmann, 1847). In this regard, the same temperature–size rule, interestingly, stipulates that the plastic phenotypic response to increased temperatures can produce smaller insects by increasing developmental rate (Atkinson, 1994). Body size and temperature relationship rules have not been corroborated and are consequently not as straightforward as theory predicts (Angilletta & Dunham, 2003). To solve this, it has been suggested that a better approach might be to generate and test theories that are tailored specifically to organisms with similar behavior and physiology (Angilletta & Dunham, 2003; DeLucia et al., 2012). Indeed, thermal response is rather the expression of the coevolution of thermal reaction norms for growth rate and size at maturity than a simplistic response that focuses on one or two mechanisms influencing life history (Angilletta et al., 2004).

Temperature has been described as a factor altering insect's body lipid and protein levels (Gligorescu et al., 2018; McCue et al., 2015). One illustrating case is that of the beetle *Ophraellla communa* whose lipid and glycogen storages decrease and increase respectively when the insect was exposed to daily phasic high temperatures (Chen et al., 2019). Changes in metabolic rates leading to anatomical and physiological alterations are the most evident expected consequences of global warming on insects (González‐Tokman et al., 2020; Sheridan & Bickford, 2011). However, combined effects of elevated temperature and carbon dioxide have been described to mitigate each other (Zvereva & Kozlov, 2006). Hence, insect body size and lipid reserves are expected to diminish (Atkinson, 1994) due to a higher metabolic rate as well as a higher total protein content produced by hydric stress and development time reduction (Papanikolaou et al., 2013). Simultaneously, fecundity is expected to increase (Huignard & Biemont, 1978), and larval development survival to decrease because of oviposition time compensation and lesser per—egg investment as females experiencing elevated temperature dispose of a shorter time window to lay eggs and harsher conditions are more likely to affect survival.

Insect responses to future climatic conditions are usually explored using the following approaches: assessment of current impacts of climatic changes based on accumulated data from the past (Andrew et al., 2013), bioassays testing climate drivers on a short‐term scale (Dyer et al., 2013), field monitoring using a geographical gradient (Hodkinson, 2005; Read et al., 2014; Slatyer & Schoville, 2016), meta‐analyses (Saban et al., 2019), and computer models predicting future scenarios (Estay et al., 2009; Northfield & Ives, 2013). Besides purely in silico models, most approaches tend to compile data to produce some predictions based on present or past conditions, which is relevant for extrapolations or climate change simulations on a short‐term scale. Despite these reasons, only a handful of investigations have used experimental designs lasting longer than 3–5 generations or explored the impacts of climate change using multigenerational experimental designs. Most of these studies have concerned marine organisms and focused on a single climate driver such as temperature (Munday et al., 2017; Shama et al., 2016), acidification or water pCO_2_ (Rodríguez‐Romero et al., 2016). One exception to these studies where temperature and pCO_2_ have been integrated is that with the marine polychaeta *Ophryotrocha labronica* (Gibbin et al., 2017). As a matter of fact, the various climate change drivers tend to offset each other's effects (Gibbin et al., 2017; Kroeker et al., 2013). Consequently, it seems reasonable to consider “climate change”, that is, increased CO_2_ concentration and temperature as a single factor.

The idea of a multigenerational selection experiment is to test the magnitude of rapid evolution. Hence, in order to discriminate whether a given phenotype is explained by plasticity or a genetic basis, a reciprocal transplant appears to be a powerful tool (Ågren & Schemske, 2012; Svensson et al., 2018). This technique was originally designed for detecting local adaptation between geographically distant populations or within a metapopulation pooling demes sharing geneflow (Blanquart et al., 2013; Kawecki & Ebert, 2004). Interestingly, some recent studies used reciprocal transplants to measure the adaptive change in a multigenerational simulation of climate change on marine species (Gibbin et al., 2017; Rodríguez‐Romero et al., 2016).

In this study, we investigated the impact of global change conditions on an insect pest's life history traits, physiology, and phenotypic plasticity. Our work is novel for the following reasons: (a) only few studies have been focused on pests' evolutionary responses to climate change; (b) we simultaneously manipulated the two driving factors of climate change; temperature and CO_2_ concentration, and (c), a multigenerational approach is used. We used the Mexican bean weevil *Zabrotes subfasciatus* Boheman as a study subject and had the following specific aims: (i) to estimate and project the modulation of the insect´s fecundity and development survival, (ii) to measure the impact of 2100 predicted climatic conditions on body size and total protein and lipid content, (iii) and to detect whether 10+ generations settles genetic adaptation or whether phenotypical plasticity is solely responsible for any measured effect on insects. Hence, we expected insects' fecundity to increase (Huignard & Biemont, 1978), and larval development survival to decrease because of oviposition time compensation and lesser per—egg investment as females experiencing elevated temperature dispose of a shorter time window to lay eggs and harsher conditions are likelier to affect survival. Simultaneously, we predicted that insect body size and lipid reserves would diminish (Atkinson, 1994) due to a higher metabolic rate as well as a higher total protein content produced by hydric stress and development time reduction (Papanikolaou et al., 2013). Finally, we also expected that genetic adaptations would be measurable by the end of the experiment and that more than phenotypic plasticity would be observed as over 10 generations have been described as more than sufficient to trigger adaptative responses (Christie et al., 2012; Laukkanen et al., 2018).

## 2 MATERIAL AND METHODS

### 2.1 Study system

The Mexican bean weevil *Zabrotes subfasciatus* Boheman (Coleoptera; Chrysomelidae; Bruchinae; Amblycerini) is a worldwide pest that affects crops and stored products of the common bean *Phaseolus vulgaris* L. This weevil is responsible for substantial agricultural damage, mostly in the New World as well as in Africa and Asia where the common bean is massively produced. The insect is sexually mature and ready for copulation immediately after emergence. Indeed, females typically lay their eggs at the very beginning of their imago life, with a peak of oviposition reached within few days (Sperandio & Zucoloto, 2004). As a capital breeding animal, *Zabrotes* adults do not feed and instead use the resources accumulated during larval development (Teixeira et al., 2009; Teixeira & Zucoloto, 2002). Consequently, females mature eggs from a limited amount of reserves and then stick them on the bean seed coat. The first instar larva will hatch and bore into the cotyledon where it will establish a larval chamber for its 30–50 days long juvenile life. A fully developed imago will emerge by cutting its way out of the seed coat.

### 2.2 Insect collection and rearing

**Figure 1:**
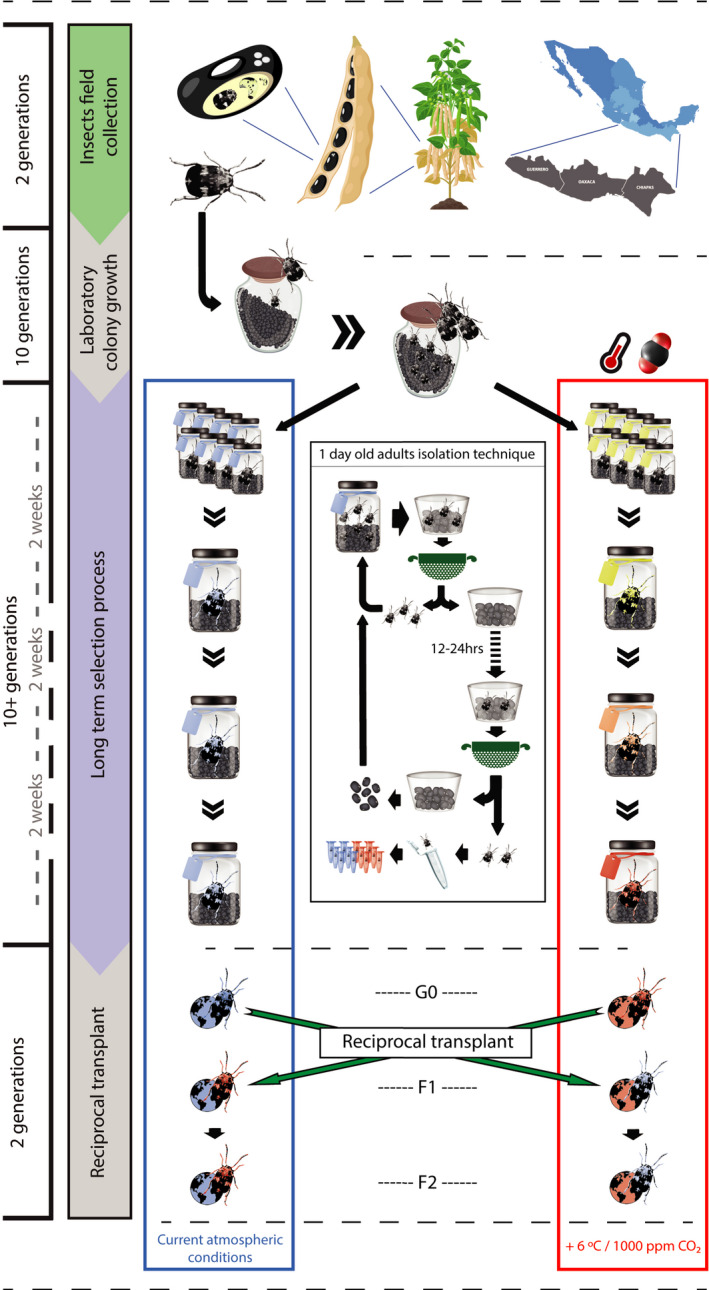
From top to bottom: Field collection of wild Zabrotes subfaciatus in the Mexican South West pacific coast, laboratory colony creation and growth during 10 generations, long‐term selection experiment with climate change simulation (2100) lines in the red box, and control lines (2017) in blue box, followed by the reciprocal experiment allowing the assessment of the genetic component of the multigenerational climate change simulation. The central black box described the technique permitting the isolation of less than one‐day‐old adult beetles for subsequent analysis and/or bioassays. The left side chronogram resumes the different phase of the whole experimental process as well as the time range in terms of generations and sampling frequency

Wild *Z. subfasciatus* were obtained from *Phaseolus lunatus* (L.) seeds collected along the South Mexican Pacific coast (Figure 1). Four locations were selected based on their relative distance (more than 15 km apart from each other) and on the number of emerging beetles (more than 15 individuals per 10 gr of wild seeds): Las Salinas (lat: 17.435301980003715, lon:‐101.19412103667855), Acapulco (lat:16.860116589814425, lon:‐99.870241926982999), Vista Hermosa (lat:16.609215969219804, lon: ‐98.483678000047803) and the experimental station of Universidad del Mar (lat: 15.922161927446723, lon: ‐97.152206227183342). The emerged wild beetles were reared in controlled environmental chambers (LD 10/14, 28°C/18°C) with random mating and no artificial selection during 10 generations prior to this study. From approximately 1,500–2,000 individuals collected from the field, 5 colonies were started by splitting the founding population in equal proportions. Furthermore, colonies were split another 2 times as the populations expanded and kept in 15 cm long side cubic glass jars each containing 2 kg of organic black bean seeds (*Phaseolus vulgaris* variety Negro Queretaro). Every 2 months, beans from all colonies were sieved, dead adults were discarded, as well as 500 g of infested seeds, and living adults were all randomly redistributed to all 20 jars with 500 g of fresh bean seeds. Despite this control of seeds, population size for all stages of our experiment cannot be calculated as beetles often hide inside the seeds.

### 2.3 Experimental colonies settings and climate change simulation

Based on the IPCC model predictions (IPCC, 2007, 2014) from 2007 to 2014 (scenario A1F1 and more recently RCP8.5), an increase of global mean temperature of 6°C and a shift of atmospheric CO_2_ concentration from 370 ppm to 1,000 ppm was selected. Since these values correspond to the worse scenario of the IPCC (2014), we decided to adopt them as the predictions from the 80's were fairly optimistic regarding the current climatic situation (Hansen et al., 1981). Two incubators (Precision Model 818, Thermo Fisher Scientific Inc) were used for the climate change simulation, a first as control (L/D: 10 hr/14 hr, 26°C/16°C with ambient air; “2017” or “present”), and a second as treatment (L/D 10 hr/14 hr 32°C/22°C mounted with an Atlas 8 digital CO_2_ controller and its flowmeter regulator [Titan controls, Vancouver, WA, USA] maintaining a constant CO_2_ air concentration of 1000 ppm [±6%], 2100” or “future”). The homogenous gas mixture inside the chambers were maintained by two 12 cm diameter fans (Essendant, Inc.). To minimize genetic drift (Rich et al., 1979), 40 replicas (starting populations) of 200 individuals (1:1 approximate sex ratio) were introduced in 15 cm long side cubic glass jars each containing 1 kg of organic black bean seeds (*Phaseolus vulgaris* variety Negro Queretaro; same variety used for the laboratory colony rearing). Each month 200 g of extra beans were added to discard competition for resources and minimizing inbreeding. Replica jars colonies were started sequentially in pairs (one replica control and one replica treatment) following the availability of freshly emerged beetles from the laboratory rearing. To minimize any internal incubator effect, all jars were randomly rearranged inside each chamber on a weekly basis.

### 2.4 Selection process monitoring

For 18 months, the colonies were monitored during the multigenerational exposure to simulated climate change and control conditions (Figure 1). Every week, a volume of 60 cm^3^ of seeds from every single jar was sieved to remove adult beetles and isolated in 5 × 5 × 4 cm plastic containers. 12 hr after, less than 1‐day‐old new imagoes emerged. This simple procedure of sieving–waiting–collecting provides younger than 1 day old, newly emerged adult beetles (Figure 1) and was used repeatedly in this study. A maximum of 10 individuals per sieved samples were randomly collected in order not to affect the population dynamics of the colonies. Every sampled insect was frozen killed and kept at −20°C until the end of the experiment. Any replica jar that would fail to provide adult insects for 3 consecutive weeks was permanently discarded.

### 2.5 Life history traits measurements

After 180 days of experimental simulation and using the same process of sieving and sampling as previously described, 1‐day‐old adult beetles were collected from each experimental jar. Males and females from the same experimental jar were randomly grouped by pairs before being deposited in 5 × 5 × 4 cm plastic boxes containing 10 bean seeds using the set.seed() function in R (R Development Core Team, 2013). Each pair was allowed to mate and lay eggs for 7 days. Each box was checked daily and any seed with eggs was removed and isolated after counting the eggs. Consequently, an emerging offspring individual could share siblings in the same seed, as well as in another seed (maximum 9 other seeds) oviposited by the same parents. Given this, replicates are nested by seed, parents and colony jar (c.f. statistical analysis). The larval development took place inside a modified 1.5 ml Eppendorf tube that was perforated 10 times with a Ø 0.7 mm needle to allow gas exchange with the controlled chamber's environment. This process was repeated every two weeks for each replica jar from day 200 to day 460. All tube‐isolated seeds were checked daily for emerging adults, which were immediately collected, frozen killed and kept at −20°C for the subsequent measurements. This protocol allowed to record larval development time, fecundity (number of eggs per pair i.e. female) and survival (number of emerging offspring/number of eggs laid per pair), and prevented the young adults from using body‐stored energy resources such as lipids before being collected.

### 2.6 Reciprocal transplant experiment

After 400 days of experimental simulation, we performed a reciprocal transplant between both 2017 and 2100 chambers. Using the same protocol for measuring life history traits described above, four experimental combinations of insect and chamber were set. However, to minimize maternal effects, F2 offspring were used to assess all measured traits. In other words, the sons and daughters of the insects emerging after the sieving process were used to provide the experimental individuals on which we performed all measurements. Our experimental groups were set as follows: (a) two control groups were arranged by introducing 2017 and 2100 insects into 2017 and 2100 chambers respectively (Figure 1); and, (b) two experimental groups. For these, a 1 day old, freshly emerged pair of adult beetles from 2017 were offered 10 seeds to oviposit and were then introduced into the 2100 chamber, whereas 2100 insects were introduced into the 2017 chamber. Each pair could oviposit for 7 days and was discarded afterward. Daily, all seeds were examined for fresh eggs, if one or more was found, then the seed presenting the egg(s) would be isolated into an individual 1.5 ml Eppendorf tube and kept in the same chamber until emergence. The remaining eggs were kept in the box until the end of the 7‐day oviposition phase. This process was repeated every two weeks until the end of the experiment (when the chambers were shut down, i.e. 240 days after the start of the reciprocal transplants). In the same manner as previously described, seeds were checked daily for emerging adults, which were immediately frozen killed and stored at −20°C for further analysis. For convention purposes and to simplify interpretation, the following terminology will be used: the 2017 chamber is referred to as “home” while the 2100 chamber is referred to as “away” for 2017 insects, and reciprocally for 2100 insects.

### 2.7 Body size, weight, total protein, and lipid measurements

The length (from the anterior end of pronotum to the posterior end of pygidium) of each bruchid beetle was measured by digital photography and pixel‐based measurement using Image J (Schneider et al., 2012) and weighted using a digital Cahn microbalance (Thermo Fisher Scientific Inc). Protein and lipid contents were assessed using a shortened version of a sequential colorimetric measurement protocol adapted for 96 well microplate assays and ELISA—type absorbance readers (Foray et al., 2012). Individuals were crushed into a single 2 ml Eppendorf tube using a steel bead and a Tissue Lyser II device (Qiagen) at 25 Hz for 30 s in 180 μl of aqueous lysis buffer solution (100 mM KH_2_PO_4_, 1 mM dithiothreitol and 1 mM ethylenediaminetetraacetic acid). After a low‐spin centrifugation of 180 g for 30 s, protein contents were measured using a simple Bradford essay (Bradford, 1976) having bovine serum albumin as standard. Absorbance at 595 nm was subsequently recorded with an Absorbance Reader ELx 800 spectrophotometer (BioTek, Inc.). Secondly, lipids were solubilized with 1,000 μl of chloroform–methanol solution (1:2 v/v) and their concentration was measured with the classic vanillin assay procedure (Van Handel, 1985). Triolein was used as standard, and absorbances were read at 540 nm using the same spectrophotometry equipment.

### 2.8 Statistical analysis

To test for an effect of climate change simulation on body size, protein content, lipid content as well as development time data over colonies age, we used linear mixed‐effects model (LMM) via restricted maximum likelihood (REML with Satterthwaite‐approximated degrees of freedom for the fixed effect), with replica jar as a random factor (Kuznetsova et al., 2017).

Regarding the life history trait approach, firstly, a GLMM (general linear mixed model) allowed us to fit by maximum likelihood (Laplace approximation) the variable development survival rate. Secondly, additional GLMM models fitted body size, protein content, lipid content, and development survival with fecundity and the number of generations, using replica jars as a random factor. The latter models were visualized as planes into a 3 days graphic representations when the models were significant.

Reciprocal transplants data were analyzed using a different approach. Primarily, for each measured trait, that is, body length, protein and lipid contents, time to emergence and fecundity, a linear mixed model including the original “home” treatment as factor for genotype (G) and the destination “away” treatment as environment factor (E) were designed in order to determine whether the variance can be attributed to genetic adaptation and assess how each trait evolved. These models were fitted by restricted maximum likelihood using Satterthwaite's method with jar replica, mother ID, and seed ID as random factors. Secondly, internal multiple comparisons were performed on least square means of the models. Regarding the predictor survival, a GLMM was performed as the data had a binomial distribution, and a Tukey (Contrasts) test permitted multiple comparisons of means in this specific case.

## 3 RESULTS

### 3.1 Life history traits

**Figure 2:**
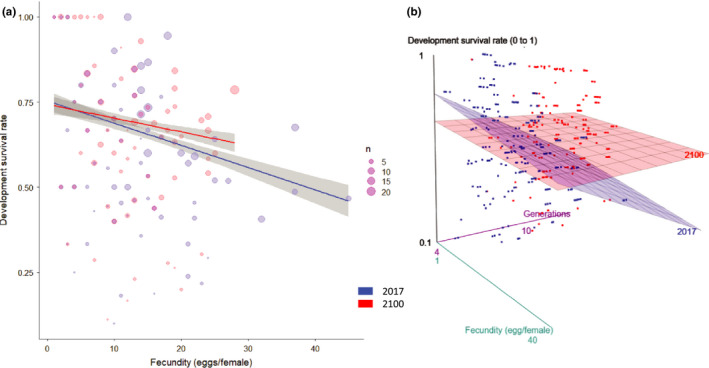
(a) GLMM model fitting survival with fecundity, dot size represents the number of overlapping data values (*n*), shaded gray area displays 95% confidence interval. (b) 3D surface plane representations of GLMM model fitting survival with fecundity over generations, blue dots and red dotes are observed values of 2017 and 2100 respectively that allowed to generate the model and their associated regression surface planes calculated from the GLMM predicted values

Fecundity alone significantly explained development survival rate (*z* = −3.175, *p* = .001496), as well as when interacting with the treatment (*z* = 3.564, *p* = .000365). Indeed, development survival decreased as fecundity increased, however, the 2100 model seemed to maintain higher probability of completing development than the control group as fecundity increased (Figure 2a).

Overall development survival rate decreased over generations in the control chamber while the treatment showed a mild increase independently of fecundity (Figure 2b). Nonetheless, when fecundity is considered, the control plane shows a stronger negative inclination as fecundity increases throughout the experiment. In other words, survival decreases strongly and significantly (Table 1) at higher fecundity for later generations. More generally, survival rate in the 2100 chamber is homogenous relatively to the control survival rates, regarding fecundity and/or the number of generations. However, neither body size, total protein, nor lipid content provided a significant model predicting development survival rate when fitted with fecundity (respectively *z* = −0.764, *p* = .44; *z* = −0.667, *p* = .505; *z* = 0.46, *p* = .65).

**Table 1:** Model outputs of GLMM fitting 1

Pooled data model				
Variables and interactions	Estimate	*SE*	*z* value	*p*
Intercept	1.54835	0.23903	6.478	<.0001
treatment	−0.57051	0.39597	−1.441	.149
fecundity	−0.03875	0.0122	−3.175	<.01
treatment fecundity	0.09042	0.02537	3.564	<.001
Model including generations				
intercept	2.756	0.646	4.264	<.0001
treatment	−2.41	1.12	−2.152	<.05
generation	−0.185	0.091	−2.046	<.05
fecundity	−0.035	0.012	−2.921	<.01
treatment generation	0.241	0.118	2.034	<.05
treatment fecundity	0.092	0.025	3.53	<.001

Note

Development survival with fecundity 2. Development survival with fecundity and generations.

### 3.2 Growth and physiological traits monitoring

**Figure 3:**
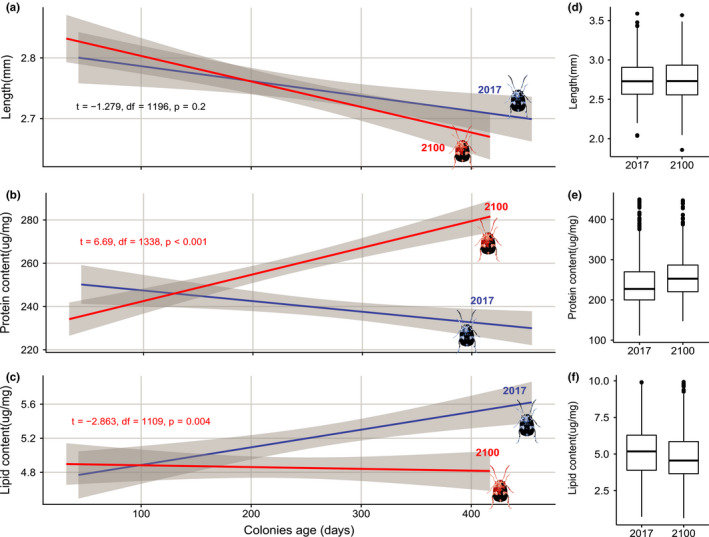
Body size (a), protein content (b), and lipid (c) content of Z. subfasciatus over colonies age, blue lines:2017 control environmental chamber group, red lines: 2100 climate change atmospheric simulation group. Shaded gray areas display 95% confidence interval bands, and (d), (e), and (f) boxplots display actual data range and distribution without the time component for plots (a), (b), and (c) respectively

**Figure 4:**
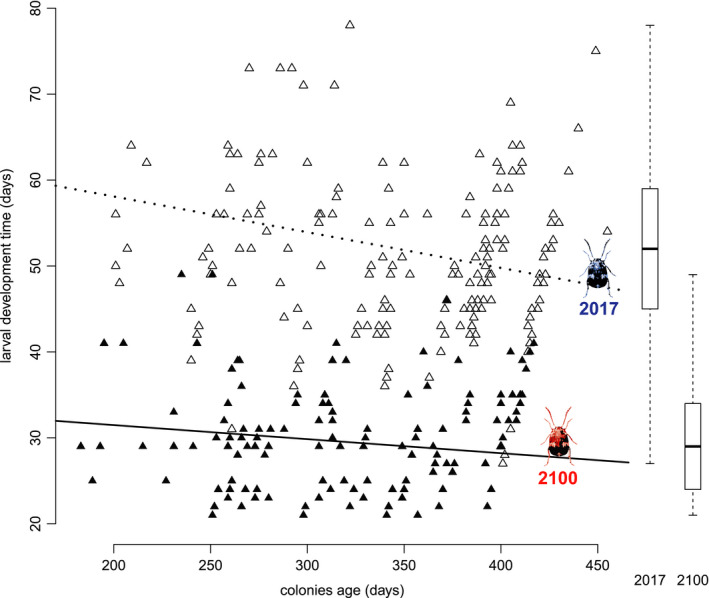
Development time of Z. subfasciatus over colonies age, empty triangles;2017 control environmental chamber group, full triangles; 2100 climate change atmospheric simulation. Dashed line is linear regression fit for 2017 data and full line is regression for 2100 data. Boxplots on the right represent distribution range of the whole dataset for both 2017 and 2100 groups

Body size did not show any significant variation during the experimental simulation (Figure 3a) despite a trend to decrease over time (*t* = −1.279, *df* = 1,196, *p* = .2). However, protein content of 2100 insects (Figure 3b) clearly increased while the control group seemed to show a mild negative slope (*t* = 6.69, *df* = 1,338, *p* < .001). Contrarily, total lipids content of the control beetles increased significantly (*t* = −2.863, *df* = 1,109, *p* = .004) in comparison to the individuals in the 2100 chamber (Figure 3c). Development time was shorter in the 2100 chamber by a factor 2 (Figure 4), and this difference was maintained during the entire experiment (*t* = −13.67, *df* = 634, *p* < .0001). An overall negative slope trend can be observed in both groups. Moreover, the variance of development time is greater in the control group (82.19) than in the 2100 group (37.45). Estimates of replica convergence show that variation of replicas variance was significant for body size and total protein but not for total lipids and the development survival rate versus fecundity data (Table 2).

**Table 2:** GLMMs Random factors output values of measured traits (Estimate of adaptation convergence)

Variance component	Variance	*p*
Body size		
Replica jar	0.00149	.01451
Residual	0.0679	
Total proteins content		
Replica jar	116.8	.00116
Residual	2,816.4	
Total lipids content		
Replica jar	0.03384	.09805
Residual	2.80623	
Development survival rate versus fecundity		
Replica jar	4.01 E−10	1

### 3.3 Reciprocal transplant experiment

**Figure 5:**
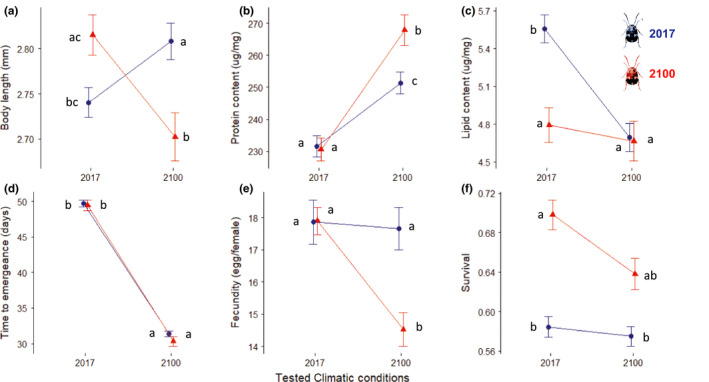
Reciprocal transplants of 2017 (blue lines and circles) and 2100 (red lines and triangles) groups bars show standard error. Blue circles in 2017 columns and red triangles in 2100 columns are controls. Small letters allow visualization of least square means multiple comparisons of Satterthwaite´s REML LMM models inside each subgraph. Exception for subgraph (f) where a GLMM was performed and a Tukey (Contrasts) test permitted multiple comparisons of means in this specific case

All statistical values and details are listed in Table 3. For all bioassays performed in this study, 5.4 individuals in average depending on the fecundity of the mother, and an average of 2.7 seeds per mother. Body size content shows the same pattern of increment when insects are exposed to the alternative chamber conditions (Figure 5a): In both cases (2017 and 2100 chambers), insect size is greater in the “away” conditions than in the “home” conditions. Both protein contents of insects from 2017 and 2100 are statistically similar in 2017 conditions, and 2100 insects show a higher content in 2100 home conditions (Figure 5b). However, insects of 2100 are richer in protein by a clear 20 μg/mg in average than insects of 2017. Lipid content dropped drastically when beetles of 2017 were exposed to 2100 conditions, but no difference was observed regarding insects of 2100 (Figure 5c). Development time from both 2017 and 2100 was similar in 2017 conditions. Similarly, both insect lines developed at the same speed in the 2100 chamber (Figure 5d). Fecundity and survival of beetles from 2017 did not show any change when transferred to the 2100 chamber, however, 2100 insects displayed a strong increase in fecundity and survival when exposed to 2017 conditions (Figure 5e,f). Regarding the variance contribution (Table 4), body size, total protein, and development time variance were explained by the environment factor only. Fecundity phenotype could not be attributed to neither genotype nor environment, while survival rate showed a significant result on genotype variance only. However, total lipid content pattern can be attributed to both genotype and environment.

**Table 3:** Models output of multiple comparisons from the reciprocal transplant experiment

Fixed effects				Random effects		
Multiple comparisons (line‐ chamber versus line‐chamber)	*df*	*t* value	Pr(>|*t*|)	Variance component	Variance	*p*
Body length						
2017–2017 versus 2017–2100	273.8	−2.4138	0.016446	Common seed	0.007005	.04613
2017–2017 versus 2100–2017	28.1	−1.9257	0.064326	Common mother	0.001306	.60885
2017–2017 versus 2100–2100	30.1	1.0246	0.313736	Replica jar	0.002223	.24604
2017–2100 versus 2100–2017	29.5	−0.2693	0.789597	Residual	0.063005	
2017–2100 versus 2100–2100	32.1	2.614	0.01352			
2100–2017 versus 2100–2100	170	2.9291	0.003866			
Protein content						
2017–2017 versus 2017–2100	251.8	−3.6565	0.0003113	Common seed	150.28	.139624
2017–2017 versus 2100–2017	24.7	0.5288	0.6016974	Common mother	309.5	.000255
2017–2017 versus 2100–2100	22.9	−4.355	0.0002335	Replica jar	96.67	.276748
2017–2100 versus 2100–2017	25.9	2.5274	0.0179403	Residual	1789.05	
2017–2100 versus 2100–2100	24.3	−2.2448	0.0341501			
2100–2017 versus 2100–2100	233.8	−5.0507	<0.0001			
lipid content						
2017–2017 versus 2017–2100	291.6	5.3709	<0.0001	Common seed	1.71E−01	.1422
2017–2017 versus 2100–2017	337.1	4.1886	<0.0001	Common mother	1.36E−09	1
2017–2017 versus 2100–2100	333.6	4.5424	<0.0001	Replica jar	3.50E−09	.987
2017–2100 versus 2100–2017	372.2	−0.4555	0.649	Residual	2.52E+00	
2017–2100 versus 2100–2100	328.3	0.1104	0.9121			
2100–2017 versus 2100–2100	361.3	0.4924	0.6227			
Time to emergence						
2017–2017 versus 2017–2100	327	24.8865	<0.0001	Common seed	17.51	<.0001
2017–2017 versus 2100–2017	29.7	0.7307	0.4707	Common mother	4.29	.009633
2017–2017 versus 2100–2100	28.7	8.1946	<0.0001	Replica jar	18.63	1.42E−11
2017–2100 versus 2100–2017	29.9	−8.0573	<0.0001	Residual	17.09	
2017–2100 versus 2100–2100	29.1	−0.7865	0.4379			
2100–2017 versus 2100–2100	248	12.0237	<0.0001			
Fecundity						
2017–2017 versus 2017–2100	656.9	0.409	0.682657	Replica jar	35.69	<.0001
2017–2017 versus 2100–2017	29.2	0.765	0.450402	Residual	47.19	
2017–2017 versus 2100–2100	28.8	2.2334	0.033461			
2017–2100 versus 2100–2017	29.4	0.6571	0.516231			
2017–2100 versus 2100–2100	29	2.1232	0.0424			
2100–2017 versus 2100–2100	663.9	3.2521	0.001203			
Fixed effects				Random effects		
	*z* value	Pr(>|*t*|)		Variance component	Variance	*p*
Survival						
2017–2100 versus 2017–2017	1.445	0.46124		glass.jar.number	4.85E−10	1
2100–2017 versus 2017–2017	4.826	<0.0001				
2100–2100 versus 2017–2017	3.586	0.00181				
2100–2017 versus 2017–2100	3.772	<0.0001				
2100–2100 versus 2017–2100	2.492	0.05822				
2100–2100 versus 2100–2017	−1.194	0.62197				

**Table 4:** Models output of reciprocal transplant experiment (Genotype × Environment)

Variables and interactions	Estimate	*SE*	*df*	*t* value	*P*
Body length					
Intercept	2.74072	0.02367	20.701	111.77	<.0001
Genotype	0.07681	0.03989	28.066	1.926	.064
Environment	0.06597	0.02733	273.814	2.414	.016
Genotype × Environment	−0.18448	0.04893	238.219	−3.771	<.001
Total proteins					
Intercept	234.617	4.769	15.497	49.199	<.0001
Genotype	−4.477	8.466	24.687	−0.529	.602
Environment	17.094	4.675	251.841	3.656	<.001
Genotype × Environment	23.027	9.224	279.856	2.496	.013
Total lipids					
Intercept	5.5642	0.1122	271.874	49.579	<.0001
Genotype	−0.7892	0.1884	337.143	−4.189	<.0001
Environment	−0.8765	0.1632	291.607	−5.371	<.0001
Genotype × Environment	0.7669	0.2767	316.837	2.771	.006
Larval development time					
Intercept	50.8982	1.2839	24.352	39.643	<.0001
Genotype	−1.4987	2.051	29.7356	−0.731	.47
Environment	−18.029	0.7245	326.975	−24.886	<.0001
Genotype × Environment	1.4426	1.4426	301.985	2.135	.034
Fecundity					
Intercept	18.2566	1.6479	26.198	11.079	<.0001
Genotype	−1.9454	2.543	29.178	−0.765	.451
Environment	−0.273	0.6673	656.941	−0.409	6.83E−01
Genotype × Environment	−3.4124	1.3151	673.855	−2.595	.009
Variables and interactions	Estimate	*SE*		*z* value	*P*
Survival rate					
Intercept	0.4839	0.134	‐	3.611	<.001
Genotype	1.4441	0.2992	‐	4.826	<.0001
Environment	0.2894	0.2003	‐	1.445	.148
Genotype × Environment	−0.7244	0.4158		−1.742	.082

## 4 DISCUSSION

In general, our results suggest that climate change alters life history strategies. For example, higher temperature and CO_2_ concentrations favored egg‐to‐adult development survival of *Z. subfasciatus*. Despite this, one would expect a higher mortality due to lesser per‐egg investment which remains true even at high female fecundity (Figure 2a). Indeed, when the component of evolutionary time is added to the model (Figure 2b), developmental survival maintains itself across generations independently of fecundity. However, it is necessary to mention that the fecundity decrease observed in the control group may be the response of laboratory selection for increased fecundity but also resulting in decreased survival.

As *Z. subfasciatus* development survival appears to increase, even in the case of high maternal fecundity, several potential explanations can be put forward. First, egg quality is greater in 2100 conditions, meaning that the ovipositing females are capable to provide more viable eggs despite the cost of laying more eggs. Second, since *Z. subfascia*tus is a capital breeder, the physiological assignment of resources during larval development is shifted from egg number to egg quality. Third, eggs and larvae simply develop better in the conditions we simulated as this species has a relatively wide temperature tolerance but a thermal optimum of 27–30°C (Sperandio & Zucoloto, 2009). In other words, our data suggest that augmented temperature and CO_2_ have the potential to lead to an increased fitness as the females seem to change their ovipositing strategy by laying fewer eggs and the developing larvae show a higher probability to reach the imago stage. Before moving on to the next section, it is important to mention that formally estimating the absence of drift is difficult as we had no total knowledge of the genetic diversity of the founder individuals used to start each colonies.

Insect body size for the 2100 simulation did not diminish throughout the experiment which is contrary to what we predicted. Rather, there was a tendency of a reduction in body size of both control and treatment groups which is likely an effect of the artificial environment. In this regard, distinct climate change drivers tend to offset each other's effects (Gibbin et al., 2017; Kroeker et al., 2013), also, body size is usually poorly described by general rules (Angilletta & Dunham, 2003; DeLucia et al., 2012) Given that our study includes CO_2_, is interesting yet not surprising to observe no body size difference between the control group and the 2100 treatment. We predict that using identical experimental designs involving either increased temperature or CO_2_ would, however, provide different outcomes. Worth mentioning is the fact that body size and temperature relationship rules are being repeatedly broken and are consequently not as straightforward as theory predicts. To fully clarify these body size responses, a better approach might be to generate and test theories using organisms with similar behavior and physiology (Angilletta & Dunham, 2003; DeLucia et al., 2012).

Protein content increased and lipid content decreased as predicted for 2100 insects. This may be a consequence of mechanisms for coping with dehydration and a different assignment of energy reserves and metabolic water. Indeed, increased metabolic flow due to higher temperature and enhanced uncoupling of mitochondrial respiration from oxidative phosphorylation, allow for greater production of metabolic water in insects in “dry environments” (Jindra & Sehnal, 1990). A similar pattern has been previously observed in a short‐term experiment using *Acanthoscelides obtectus*, a bruchid beetle species close to *Z. subfasciatus,* when temperature increased from 20 to 30°C (Sönmez & Gülel, 2008), but no relevant explanation was found regarding the context of our multigenerational simulation. Consequently, as triglycerides yield almost two times more metabolic water than glycogen (Arrese & Soulages, 2010), fat storage would have been compromised in a dryer environment during larval development. Hence, the pupation process might have further reduced the fat storage through metabolic water extraction as the insect stops feeding and experiences costly transformations in dryer air conditions. This is coherent with findings in other insects. For example, the tsetse fly *Glossina* spp. uses lipid storage during pupation for water balance control depending on the ambient humidity and temperature. Moreover, fat consumption increases with the temperature while the pupal period reduces (Bursell, 1958, 1960; Kleynhans & Terblanche, 2009). Regarding the clear positive slope of protein content in 2100 group, one explanation is that it shows desiccation and increasing tolerance to desiccation. As the experiment progressed, it is likely that individuals from the 2100 group achieved a greater tolerance to warmer and dryer conditions. Possibly these animals afforded to be functional with less water in their environment and body as the lipid content stabilizes over time. This is in agreement with studies in *Drosophila melanogaster* which were selected for increased desiccation resistance (Telonis‐Scott et al., 2006),

While studies on heat shock proteins and other temperature stress‐related processes are common (Adamo, 2012; Sørensen & Loeschcke, 2007; Sørensen et al., 2005; Wang et al., 2014), very scarce data are available on total protein and lipid contents. In a similar way, numerous studies have provided evidence of, for example, the indirect role of CO_2_ through plant tissue alteration (Cornelissen, 2011; Knepp et al., 2005; Murray et al., 2013; Xu et al., 2019) or behavioral approaches on hematophagous insects using CO_2_ as a pointer (Guerenstein & Hildebrand, 2008; Jones, 2013; Lazzari et al., 2013; McPhatter & Gerry, 2017). Despite this, literature on direct effects of CO_2_ is scarce, aside from studies testing extreme cases such as over 20% of CO_2_ (Mitcham et al., 2006), as it has been considered that direct metabolic effects of CO_2_ on herbivorous insects are insignificant when the effect on the plant is removed (Coviella & Trumble, 1999). However, laboratory studies using CO_2_ anesthesia on *Drosophila melanogaster* show that metabolic changes persist 14 hr after acute CO_2_ exposure (Colinet & Renault, 2012; Nilson et al., 2006). This fact should be considered to understand that a prolonged exposure to doubled or tripled CO_2_ air concentration may impact insect physiology.

The multigenerational simulation of climate change conditions provided a clear contrasting pattern between anatomical and physiological data. As suggested by literature (Angilletta & Dunham, 2003), body size and temperature were not tightly associated by the temperature–size rule (Atkinson, 1994) as no difference was observed between 2017 and 2100. This would fit with a recent metanalysis that explored the species‐specificity of the temperature–size rule (Klok & Harrison, 2013). This study indicated that large interspecific variation is either explained by strong interactions with nutrition, or selection based on microclimatic or seasonal variation not captured in classic macro‐environmental variables. Indeed, the clear patterns of protein content increase and lipid decrease (relatively to control) imply that metabolic changes are undergoing while the overall exoskeleton size remained unchanged. Interpreting the body size and total protein data must be done with the knowledge that not all replicas converged toward the same outcome as they did for the other measured traits. A small initial population size could have generated such a phenomenon, but some traits converged while others did not, suggesting a greater starting effective population for future experimental designs.

Besides the anticipated faster growth in elevated temperature, an interesting pattern of reduced variance was observed in the development time data. This pattern is actually coherent with previous bioassays (Marinho et al., 2015) and models (Régnière et al., 2012) addressing the impact of temperature on this parameter. Indeed, development time variance tends to shrink at warmer temperatures only if a metabolic and/or physiological threshold is not reached or verged upon (Régnière, 1987). A narrower temporal phenology might reduce variability in ecosystems and agrosystems and is also prone to desynchronize plants–herbivores–predators. Another aspect that should be considered for further investigation is that development time can be offset by temperature fluctuation range (Xing et al., 2015) and a greater climatic, hence, temperature stochasticity is expected with global warming predictions (IPCC, 2007, 2014). Therefore, longer development time and larger variances are expected under a more realistic climate variability simulation.

The overall pattern leads to hypothesize that a pest insect such as *Z. subfasciatus* could in fact be advantaged when facing elevated temperature and CO_2_ levels. Bean seeds providing a micro‐environment, the growing larvae are virtually affected by a highly similar number of factors in the field and in a laboratory colony, aside from, of course, parasitoid attacks which are common predators in this system (Schneider & Córdoba‐Aguilar, 2019a). Given that the young beetles spend over one month encapsulated into their seed, larvae are protected from most physical factors such as atmospheric and mechanical damages. However, Chacidoids and Braconids parasitoid wasps would obviously be able to attack the protected larvae inside the bruchid larval chamber and then constrain population growth (Aebi et al., 2008; Schneider & Córdoba‐Aguilar, 2019b).

Our reciprocal transplant experiment produced interesting patterns of phenotypic variation. However, genetic adaptation can be attributed to the variance of the total lipid content only, despite our expectations that 10+ generations would generate such adaptation in most measured traits. Body size is the only trait displaying an expected pattern of “local adaptation”. However, this pattern indicates an increase of body size when the insects are transplanted, independently of their origin (2017 or 2100). Nonetheless, this is consistent with the aforementioned theory and literature conjecturing that both elevated temperature and CO_2_ concentration might mitigate their effects reciprocally (Zvereva & Kozlov, 2006). On one hand, it seems extremely counterintuitive that body size when transplanted, especially in the case of 2017 insects that are supposedly more constrained in the challenging 2100 conditions. On the other hand, one would expect the 2100 acclimated beetles to have a better fitness in more supposedly optimal conditions.

Unfortunately, body size variation as well as protein, time to emergence, and fecundity variances are not explained by a genetic component. Moreover, the development time data confirms that the thermal difference amplitude between both chambers is such an extent that the metabolic rate is irrelevant for detecting genetic change. Indeed, the phenological response to temperature is literally masking off any potentially measurable difference between transplants and control groups in each conditions (Figure 5), due to the fact that insect's development time is tightly connected to temperature (Damos & Savopoulou‐Soultani, 2012). The total lipid phenotypes recorded in the reciprocal transplant experiment is the only case in our data where the measured trait variance can be properly attributed to genetic adaptation. Unfortunately, one would expect the lipid levels of 2100 insects to be higher when exposed to their home conditions. Typically, insects experimenting their optimal environmental conditions present optimal energy storage levels (Arrese & Soulages, 2010; Klepsatel et al., 2019). Consequently, this pattern cannot be associated to the idea that the selected lines are fitter under home conditions and then forbid affirming that *Z. subsfasciatus* as an organism is adapted to 2100 conditions. Nonetheless, it is well known that different organs, pathways, and genes evolve at different speeds (Gillespie, 1986; Wilke, 2004; Zhang & Townsend, 2009), therefore, it is safe to hypothesize that lipid metabolism is under selection.

The puzzling finding that fecundity and development survival of 2100 beetles are lower under their home conditions could be explained by the cost of thermal tolerance plasticity. When an organism is being thermally challenged, either by colder, warmer or highly variable temperatures, thermal tolerance plasticity tends to vary whether this organism is adapted to cold, warm, stable or instable temperatures (Angilletta, 2009; Brahim et al., 2019; MacLean et al., 2019). In our study, it is premature to attribute higher thermal tolerance plasticity to the resilience of 2017 insects or to the adaptability of 2100 insect's fecundity and survival when exposed to different conditions. Therefore, if the lipid data is added to the interpretation, we could hypothesize that 2017 and 2100 insects present different cost when handling strategies for maintaining fitness in “away” conditions. On one hand, the 2100 insects adjust fecundity and survival to their lower fat storage: colder conditions are less costly in terms of heat resistance. Additionally, their metabolism is already able to handle high temperatures with minimal energy requirements. On the other hand, 2017 insects respond to heat with high energy coping mechanisms using their greater stock of lipids (González‐Tokman et al., 2020).

Plasticity is usually expected to enable organisms to cope with fast‐changing environments (Gienapp et al., 2008). However, even though plasticity mostly occurs within a generation, it has been reported that the conditions experienced by one generation could interact with the conditions experienced by the subsequent generations (Donelson et al., 2018). This phenomenon is known as transgenerational plasticity (TGP), which is likely to take place in a reciprocal transplant experiment as logistics do not fully discard maternal and paternal effects as well as epigenetic transmission (Donelson et al., 2018; Shama et al., 2016). In fact, we cannot discard maternal effects due to the ovipositing behavior (several eggs per seed and on several seeds) of *Z. subfasciatus*, we should discuss our results consistently with this fact. Hence, we cannot exclude TGP as the outcome of our study. It is true that *Zabrotes*, being a worldwide spread multivoltine pest, should have a great potential for phenotypic plasticity (Aebi et al., 2008; Alvarez et al., 2006; Cuny et al., 2017). Consequently, the experiment should be performed for a greater number of generations. Ideally a similar design could be easily implemented using faster developing insects such as *Drosophila spp*. or sister genders causing economic damage such as *Rhagoletis*. Another approach would be to use full genome transcriptomics, as mentioned previously, or even more modern tools such as “Evolve and resequence” (Schlötterer et al., 2015) to pinpoint which genes or gene cluster regulations are affected by increased temperature and CO_2_.

## 5 CONCLUSION

The role of life history traits plasticity and evolution has been overlooked in climate change ecology (Donelson et al., 2018; Lancaster et al., 2017). This fact is probably explained by the lack of multigenerational experimental data. Our study provides such data and helps guiding the way to more realistic predictions in climate change biology. Moreover, it informs that elevated temperature and CO_2_ together affect the physiology, life history traits and the evolutionary direction of a laboratory raised colony of *Zabrotes subfaciatus*. So far, it seems that this pest will deal with climate change by adjusting mainly survival and physiological traits. Future research should look at whether such changes imply higher costs for plant productivity and thus risks for food security.

## CONFLICTS OF INTERESTS

None declared.

## AUTHOR CONTRIBUTIONS

**David Schneider:** Conceptualization (lead); data curation (lead); formal analysis (lead); investigation (lead); methodology (lead); project administration (equal); resources (equal); software (equal); supervision (equal); validation (equal); visualization (equal); writing–original draft (lead); writing–review and editing (equal). **Alejandra G. Ramos:** Formal analysis (supporting); software (equal); validation (equal); writing–original draft (supporting); writing–review and editing (equal). **Alex Córdoba‐Aguilar:** Conceptualization (equal); funding acquisition (lead); investigation (equal); supervision (lead); validation (equal); writing–original draft (equal); writing–review and editing (equal).

## Data Availability

Multigenerational experimental simulation data & reciprocal experiment data: Dryad https://doi.org/10.5061/dryad.h44j0zph5.
